# A Proposed Bill to Regulate the Practice of Veterinary Surgery, Medicine, and Dentistry in the State of Colorado

**Published:** 1897-01

**Authors:** 


### LEGISLATION.

### A Proposed Bill to Regulate the Practice of Veterinary Surgery, Medicine, and Dentistry in the State of Colorado.

*Be it enacted by the General Assembly of the State of Colorado:*

Section 1. A Board is hereby established to be known as the State Board of Veterinary Examiners. Said Board shall be composed of three practising veterinary surgeons who are graduates of some recognized but different colleges of veterinary surgery and medicine, which Board shall be appointed by the Governor within thirty days after this Act becomes a law. Said Board shall be appointed, one for two years, one for four years, and one for six years. Thereafter the Governor shall appoint biennially one member of said Board possessing the qualifications before stated. In the event of resignation, death, or removal of any member or all the members of said Board, the Governor shall appoint their successors within thirty days.

Sec. 2. The Board of Veterinary Examiners shall elect from its members a president, a secretary, and a treasurer, and may adopt such rules as they may deem proper for the performance of their duties and to carry into effect the provisions of this Act. They may also adopt a seal, which shall be affixed to all certificates issued by them, and the president and secretary shall sign all such certificates, but that no member of the Board of Veterinary Examiners shall require or accept any fee for services rendered in his capacity as such member of the Board of Veterinary Examiners except their actual travelling and necessary expenses.

Sec. 3. It shall hereafter be unlawful for any person to practise veterinary surgery, medicine, or dentistry, or any branch thereof in the State of Colorado without having previously obtained a certificate from the Board of Veterinary Examiners or having registered as hereinafter provided.

Sec. 4. Applicants for certificates to practise veterinary surgery, medicine, or dentistry, or any branch thereof in the State of Colorado shall file their application with the Secretary of the Board of Examiners. If the applicant be a graduate of a recognized college of veterinary surgery and medicine, he shall present his diploma to the Board or give satisfactory evidence of his being a graduate of a recognized college of veterinary surgery and medicine. The Board of Examiners shall examine all diplomas or evidences of such as to their genuineness.

SEC. 5. But it is further expressly provided that any person who shall have gained his livelihood solely from the practice of veterinary surgery and medicine as a profession in this State for the past five years immediately preceding the passage and approval of this Act shall be permitted to continue the practice of said veterinary surgery and medicine if he shall within ninety (90) days after the passage and approval of this Act register his name and address with said Board of Veterinary Examiners and give satisfactory proof to said Board of Examiners that he has practised in the State of Colorado for five years and is qualified to register under the provisions of this Act.

SEC. 6. All persons commencing the practice of veterinary surgery, medicine, or dentistry, or any branch thereof in the State of Colorado after the passage and approval of this Act shall be graduates of a legally authorized and recognized veterinary school, college, or university, and shall comply with the provisions of this Act.

SEC. 7. Upon the approval of diploma or satisfactory evidence that applicant is entitled to register under the provisions of this Act said Board of Veterinary Examiners shall issue to such applicant a certificate to practise veterinary surgery, medicine, or dentistry, or any branch thereof in this State, and said certificate shall be conclusive as to the rights of the lawful holder thereof to practise veterinary surgery, medicine, or dentistry in the State of Colorado, provided that said Board shall have power to reject any applicant for registration who is not fully qualified under the provisions of this Act.

SEC. 8. There shall be paid to the Treasurer of said Board of Examiners in advance a fee of five dollars ($5) for each certificate issued to graduates of veterinary colleges, and a fee of ten dollars ($10) by all applicants for registration under Section 5 of this Act; and in case of failure of approval said fee shall be forfeited to the Board of Veterinary Examiners.

SEC. 9. It shall be the duty of said Board of Examiners to keep a register of all practitioners qualified under the provisions of this Act and to register the name, age, and time spent in the study and practice of veterinary surgery, medicine, or dentistry, and, if a graduate, the name and location of the school or college granting his diploma. Such records shall be *prima facie* evidence of all matters therein recorded, and shall be open to public inspection at all times, within reasonable hours, at the office of the Secretary of the Board. Such register shall be turned over to each succeeding Board of Examiners.

SEC. 10. The President of said Board shall have the power to administer oath, and said Board the authority to take testimony in all matters relating to its duties.

SEC. 11. Every person holding a certificate under the Board of Veterinary Examiners shall have the same recorded in the office of the clerk of the county wherein he resides.

SEC. 12. Any person practising veterinary surgery, medicine, or dentistry, or any branch thereof in the State of Colorado without complying with the provisions of this Act shall be deemed guilty of a misdemeanor, and upon conviction thereof shall be punished by a fine of not less than $50 nor more than $300, or by imprisonment in the county jail for not less than ten days nor more than sixty days, or by both fine and imprisonment, for each and every offence; and all fines collected in accordance herewith shall be paid to the Treasurer of said Board of Veterinary Examiners.

SEC. 13. Any person shall be regarded as practising veterinary surgery, medicine, or dentistry, or any branch thereof within the meaning of this Act who shall publicly profess to be a veterinary surgeon or prescribe for sick and injured animals and accept fee for such services, or attach to his name the title "V.S." or "Veterinary Surgeon," or any veterinary title in a medical sense.

But nothing in this Act shall be construed to prohibit gratuitous services in a case of emergency.

SEC. 14. All moneys received by the Treasurer of said Board of Examiners shall be paid by him to the State Treasurer, and all expenses of said Board shall be paid out of this fund upon warrants drawn by the State Auditor on the State Treasurer and signed by the President and Secretary of the Board of Examiners, and no other money shall be paid out of the State Treasury for the use and purpose of this Act, provided that any excess in this fund above the expenses of said Board of Examiners shall stand to the credit of said Board of Examiners.

SEC. 15. The said Board shall meet as a Board of Examiners in the city of Denver on the second Monday in January and July of each and every year, and at such other times and places as they may find necessary for the performance of their duties.

An extremely important decision has just been rendered by the Supreme Court of Minnesota bearing upon legislation as to the powers of Boards of Health in enforcing ordinances regulating the production and sale of milk. The court held that this ordinance was not extraterritorial, and that it is competent for a city council to require that an applicant for a license to sell milk within the city shall consent that the dairy herd from which he obtained his milk may be inspected by the Commissioner of Health of the city, although such dairy herd is kept outside the city limits.

"The tuberculin-test" held not unreasonable. The requirement that he shall consent, as a condition precedent to obtaining such license, that the animals from which he obtains the milk shall be subjected to the tuberculin-test, is not unreasonable.

As to whether a license from a city under an ordinance from Councils is a substitute or supplemental to a license from the State Dairy Commissioner is not decided, but in either view the ordinance is authorized by the Act of 1895.

The points involved in this decision are such that every veterinarian should be familiar with them, as they go to the very core of this whole question of proper safeguards to consumers of milk.

A bill will be introduced in the Legislature of Delaware to pay from the State Treasury all expenses incurred in sending poor children bitten by rabid dogs to the Pasteur Institute in New York.

In New Hampshire the proposed Act requires registration of all veterinarians after July 1, 1897. All registrations after satisfactory examination before an appointed board of three examiners. Subjects: anatomy, physiology, materia medica and therapeutics, practice of medicine and surgery. Examiners to be members of the State Association. First examination fee, ten dollars; subsequent ones within two years. Two examinations yearly. Fees to pay expenses of board. Certificates not to permit the use of any degree unless formerly obtained from a veterinary college. Exemption clause for those graduates who have been in active practice for three years prior to date of application for régistration.

---

A cavalry troop, consisting of forty of the leading young business men and prominent citizens of Des Moines, Iowa, has been organized, and Gen. Lincoln, of the Iowa Agricultural College, drills them once a week. Their horses include some very good Missouri and Kentucky saddlers, and the interest that is being created promises to stimulate greatly the demand for gaited saddlers in Des Moines.

